# Effects of BPZ and BPC on Oxidative Stress of Zebrafish under Different pH Conditions

**DOI:** 10.3390/molecules27051568

**Published:** 2022-02-26

**Authors:** Ying Han, Yumeng Fei, Mingxin Wang, Yingang Xue, Yuxuan Liu

**Affiliations:** 1 School of Environmental & Safety Engineering, Changzhou University, Changzhou 213164, China; feiyumeng1997@163.com (Y.F.); yzxyg@126.com (Y.X.); dinoice0401@163.com (Y.L.); 2 Jiangsu Engineering Research Center of Petrochemical Safety and Environmental Protection, Changzhou 213164, China

**Keywords:** bisphenol analogs, zebrafish, oxidative stress

## Abstract

To further understand the toxic effects of bisphenol Z (BPZ) and bisphenol C (BPC) on aquatic organisms, zebrafish (*Danio rerio*) were exposed to 0.02 mg/L BPZ and BPC mixed solution in the laboratory for 28 days. The impacts of BPZ and BPC on the activity of the antioxidant enzymes, expression of antioxidant genes, and estrogen receptor genes in zebrafish under different pH conditions were studied. The changes of glutathione peroxidase (GSH-Px), reduced glutathione (GSH), total superoxide dismutase (T-SOD), catalase (POD), and malondialdehyde (MDA) in the zebrafish were detected by spectrophotometry. The mRNA relative expression levels of CAT, GSH, SOD, ER*a,* and ERb1 in the experimental group were determined by fluorescence quantitative PCR. The results showed that SOD activity and MDA content were inhibited under different pH conditions, and the activities of GSH, GSH-Px, and POD were induced. The activities of POD and GSH induced in the neutral environment were stronger than those in an acidic and alkaline environment. The mRNA relative expression levels of SOD and GSH were consistent with the activities of SOD and GSH. The mRNA relative expression levels of CAT were induced more strongly in the neutral environment than in acidic and alkaline conditions, the mRNA relative expression levels of ER*a* were induced most weakly in a neutral environment, and the mRNA relative expression levels of ER*b*1 were inhibited the most in a neutral environment.

## 1. Introduction

Bisphenol analogs (BPs) are a class of environmental endocrine disrupts with a similar structure to bisphenol A (BPA), which are widely found in various environmental media [1]. On 18 October 2008, Canada was the first country to declare BPA a toxic substance, banning its use in baby bottles and restricting its use in all food packaging and containers in 2010. This was followed by policies to restrict the use of BPA around the world [2]. To meet the needs of the industrial market, the production and use of BPA substitutes have gradually increased, such as bisphenol C (BPC) used in flame retardant preparation and bisphenol Z (BPZ) applied in chemical compounds manufacture [3]. The new double phenol compounds in the process of production, use and waste treatment, etc., also inevitably enter the environment especially through water (more than 90%, water solubility is related to pH values), causing harm to the aquatic environment, ecological situations, and human health [4,5,6,7]. 

Zebrafish (*Danio rerio*) is an important vertebrate model organism. It is named because of the zebra-like stripes on its side and the length of adult fish is 3–5 cm [8]. Zebrafish have organs and systems similar to those of mammals, such as nerves, digestion, reproduction, immunity, endocrinology, and cardiovascular diseases. Therefore, zebrafish were mainly used in studies related to neurodevelopment and genetics at first [9]. Similarly, zebrafish is widely used in environmental toxicology studies and can be used as an important tool to detect the toxicological effects of bisphenol compounds [10]. 

Oxidative stress refers to the physiological and pathological reactions of cells and tissues caused by the production of reactive oxygen species (ROS) and reactive nitrogen species (RNS) in the body under the harmful stimulation of the internal and external environment. At present, it has become one of the important topics in environmental toxicology research [11]. Although the number of studies on BPA analogs is limited, according to existing research reports, BPA analogs can cause cytotoxicity, reproductive toxicity, neurotoxicity, and endocrine disruption [12,13]. Ullah et al. conducted in vivo experiments on rats and found that exposure to BPA, bisphenol B (BPB), bisphenol F (BPF), and bisphenol S (BPS) for 28 days would cause ROS production and LPO activity in rat sperm and lead to DNA damage in rat sperm [14]. Park et al. conducted experiments on the acute toxicity, vital parameters, and defense bodies of marine rotifers and found that exposure to BPA, BPF, and BPS significantly increased intracellular ROS levels and glutathione S-transferase (GST) activity [15]. Wu et al. reported short-term exposure to BPA and nonylphenol inhibited the contents of total glutathione (TG), reduced glutathione (GSH), oxidized glutathione (GSSH), catalase (CAT), superoxide dismutase (SOD), glutathione peroxidase (GSH-Px), glutathione reductase (GR), glutathione S-transferase, and other antioxidant enzymes in serum of zebrafish embryos [16]. 

In this study, the toxic mechanism of BPC and BPZ on zebrafish was explored by detecting the expression levels of oxidative stress-related genes and enzyme activities under different pH conditions. The aim of this study was to provide toxicological data for the toxicity of BPC and BPZ on zebrafish and possible impacts on aquatic organisms.

## 2. Results and Discussion

### 2.1. Effects of a Mixed Solution of Bisphenol C (BPC) and Bisphenol Z (BPZ) on the Expression of Oxidative Stress-Related Genes and Estrogen Receptor Genes in Zebrafish

#### 2.1.1. Effects of Phase I Mixed Solution on the Expression of Oxidative Stress Gene and Estrogen Receptor Gene in Zebrafish

**Figure 1:**
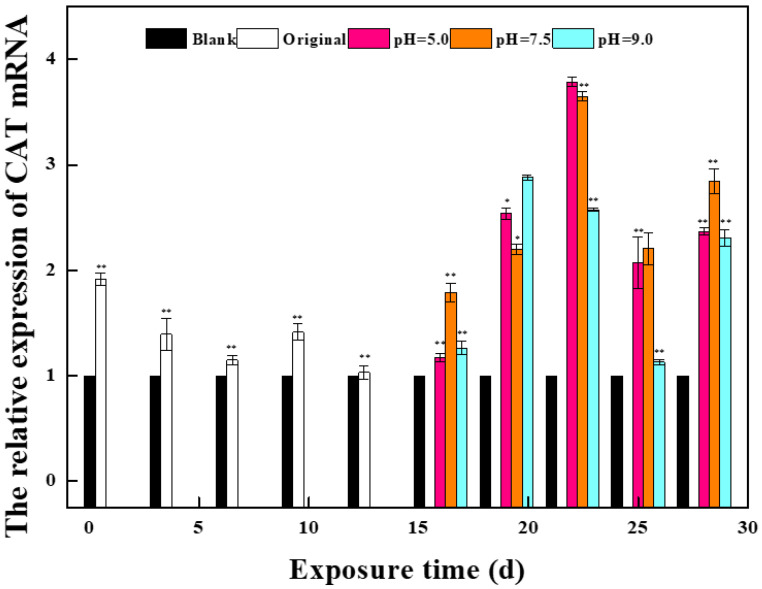

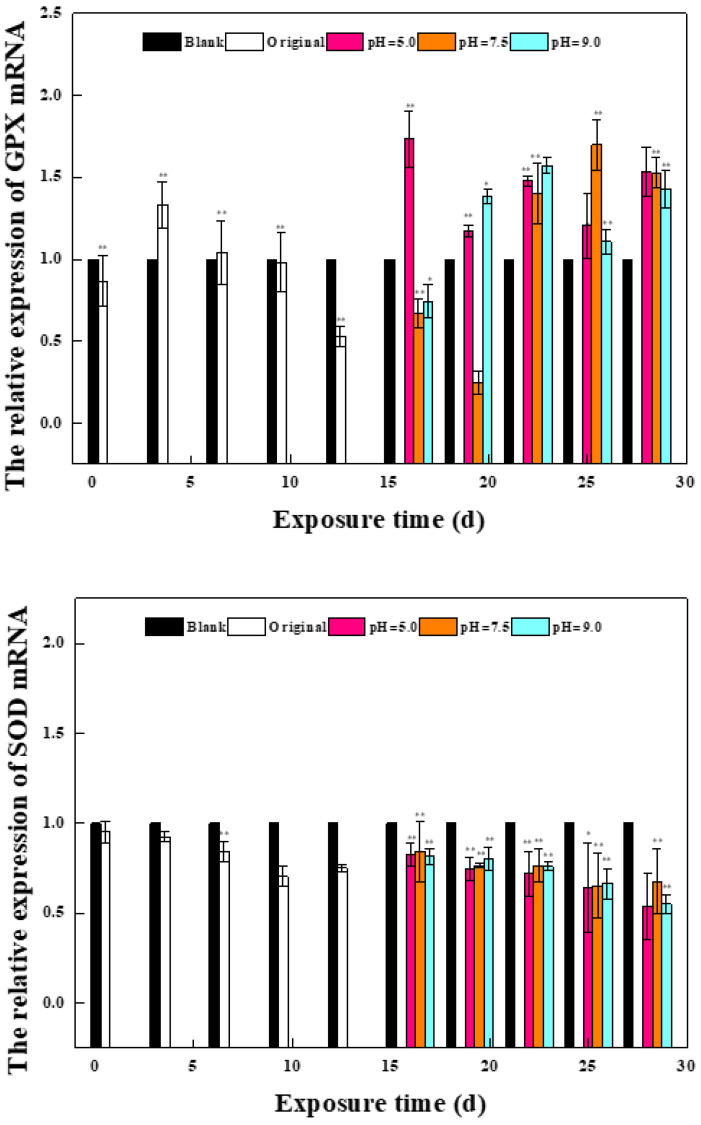

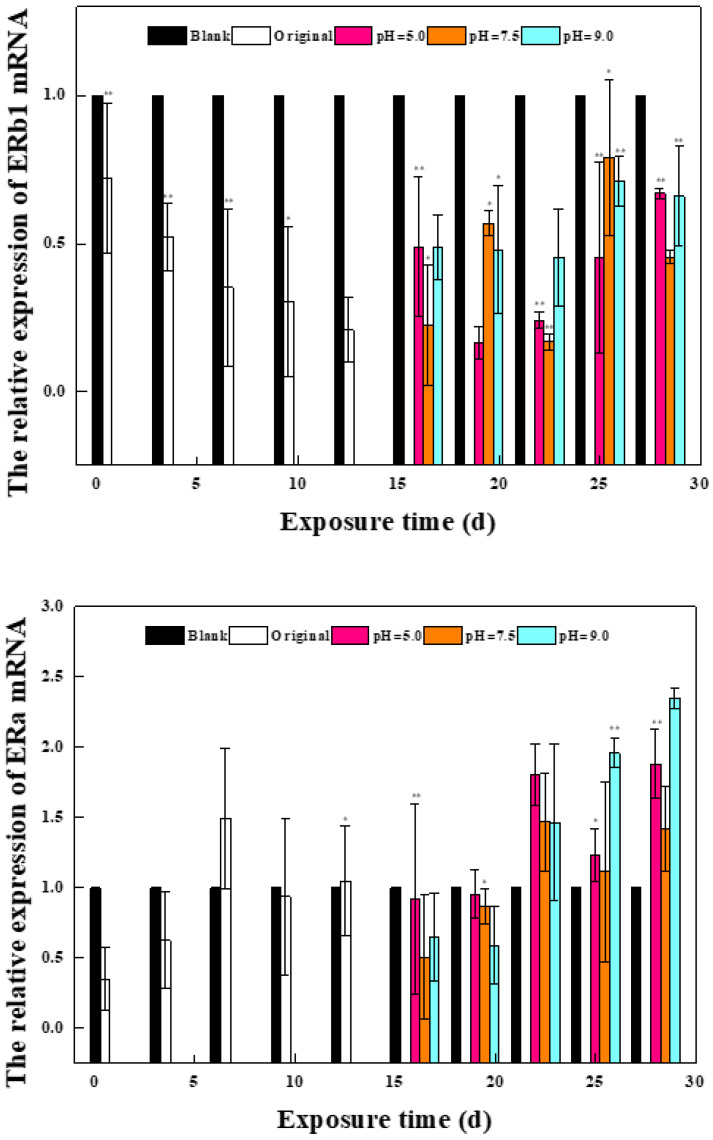
Effects of BPC and BPZ on mRNA expression levels of oxidative stress-related genes in zebrafish (*n* = 3) (Note: 0.01 < * *p* < 0.05; ** *p* < 0.01, compared with the control group).

BPC and BPZ can change the expression levels of related genes in the body’s antioxidant defense system. Therefore, this study selected 3 antioxidant genes and 2 estrogen receptor genes as references to reveal the mechanism of the oxidative stress effect of bisphenol estrogen on zebrafish from the transcriptional level. It can be seen from Figure 1 that the expression levels of antioxidant-related genes and estrogen receptor genes in zebrafish are disturbed to varying degrees. Compared with the blank control group, the relative expression level of the CAT gene was significantly upregulated to 1.9 times on day 1 (*p* = 0.0004; *p* < 0.01). In the following 12 days, although the relative expression level decreased, it was still upregulated compared with the blank control group. The relative expression level of the GPX gene was upregulated to 1.3 times at day 4, and compared with the blank control group at other periods except day 4, the relative expression level of the GPX gene was downregulated, showing great overall fluctuation. The relative expression level of the SOD gene was reduced to 0.7 times on the 10th day, which showed an overall downregulation state compared with the blank control group. The relative expression level of the ERb1 gene decreased to 0.2 times on day 13, showing a time-effect relationship. The relative expression level of the ER*a* gene was upregulated to 1.5 times at day 7 and down to 0.4 times at day 1, indicating a wide fluctuation.

#### 2.1.2. Effects of a Mixed Solution of the Second Stage on the Expression of Oxidative Stress Gene and Estrogen Receptor Gene in Zebrafish

In the second stage of exposure, the nine parallel groups in the first stage were divided into three groups with different pH values, and the related gene detection method was the same as in the first stage. It can be seen from Figure 1 that the expression levels of antioxidant genes and the expression of estrogen receptor genes in zebrafish of the three groups with different pH were different. Compared with the blank control group, the relative expression levels of CAT genes in the three groups were upregulated in the first 14 days, which was consistent with the development trend of the first 13 days. The relative expression of the CAT gene was upregulated to the maximum value on day 22 in the low pH group, to 3.8 times on day 22 in the low pH group, and to 3.7 times on day 22 in the middle pH group. The relative expression of the CAT gene in the high pH group reached the maximum value on day 19, which was 2.8 times higher than that in the blank control group. Compared with the blank control group, the relative expression of the GPX gene in the low pH group was upregulated to the maximum value on day 1 and then decreased, but it was still upregulated compared with the control group. The relative expression of the GPX gene in the middle pH group increased to the highest value on day 25, which was 1.7 times that in the control group, and decreased to the lowest value on day 19, which was 0.2 times that in the control group. The relative expression of the CAT gene in the high pH group reached the highest value on day 22, which was 1.6 times that in the control group. Although the relative expression levels of the GPX gene in the three groups fluctuated differently, the final results tended to be consistent. The relative expression of the SOD gene in the second stage was decreased compared with that in the blank control group, which was consistent with that in the first stage, and the fluctuation of the three groups was similar. Wu et al. exposed zebrafish embryos to different concentrations of BPA and nonylphenol, finding that GSH, SOD, and other genes’ activities were significantly suppressed. The oxidative stress was onset, which is in accordance with the results of this study [16].

The relative expression of estrogen receptor gene ER*b1* was downregulated in all three groups compared with the blank control group, which was consistent with the results of the first stage. The relative expression level of the ER*b1* gene in the low pH group decreased to the lowest value on day 19, and the relative expression level of the ER*b1* gene in the middle and high pH group decreased to the lowest value on day 22. Compared with the blank control group, the relative expression level of ER*a* in all three groups was upregulated, which was contrary to the results of the first stage. The relative expression of the ER*a* gene in the low and high pH groups was upregulated to the highest value on day 28, which was 1.9 and 2.3 times that in the control group, respectively. The relative expression of the ER*a* gene in the middle pH group was upregulated to the maximum value at day 22, which was 1.4 times that of the control group.

### 2.2. Effects of a Mixed Solution of BPC and BPZ on the Expression of Oxidative Stress-Related Genes and Estrogen Receptor Genes in Zebrafish

#### 2.2.1. Effects of Mixed Solution at the First Stage on Antioxidant Enzyme Activity and MDA Content in Zebrafish

BPC and BPZ can disbalance the oxidation system and antioxidant system of the body, resulting in excessive production of reactive oxygen species (ROS) and other free radicals in the body. The oxidation degree exceeds the ability of cells to remove oxides themselves, and a large number of oxidation intermediates are produced, leading to oxidative damage of the body.

**Figure 2:**
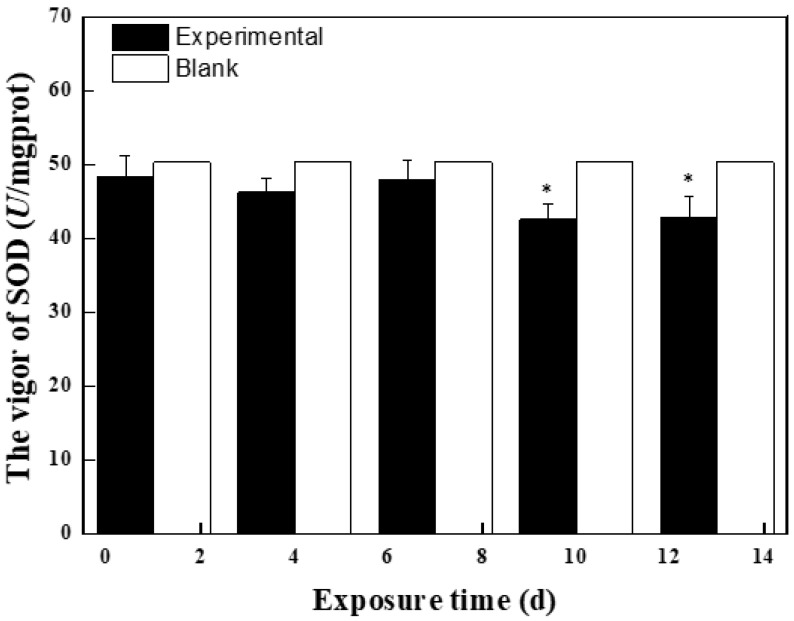

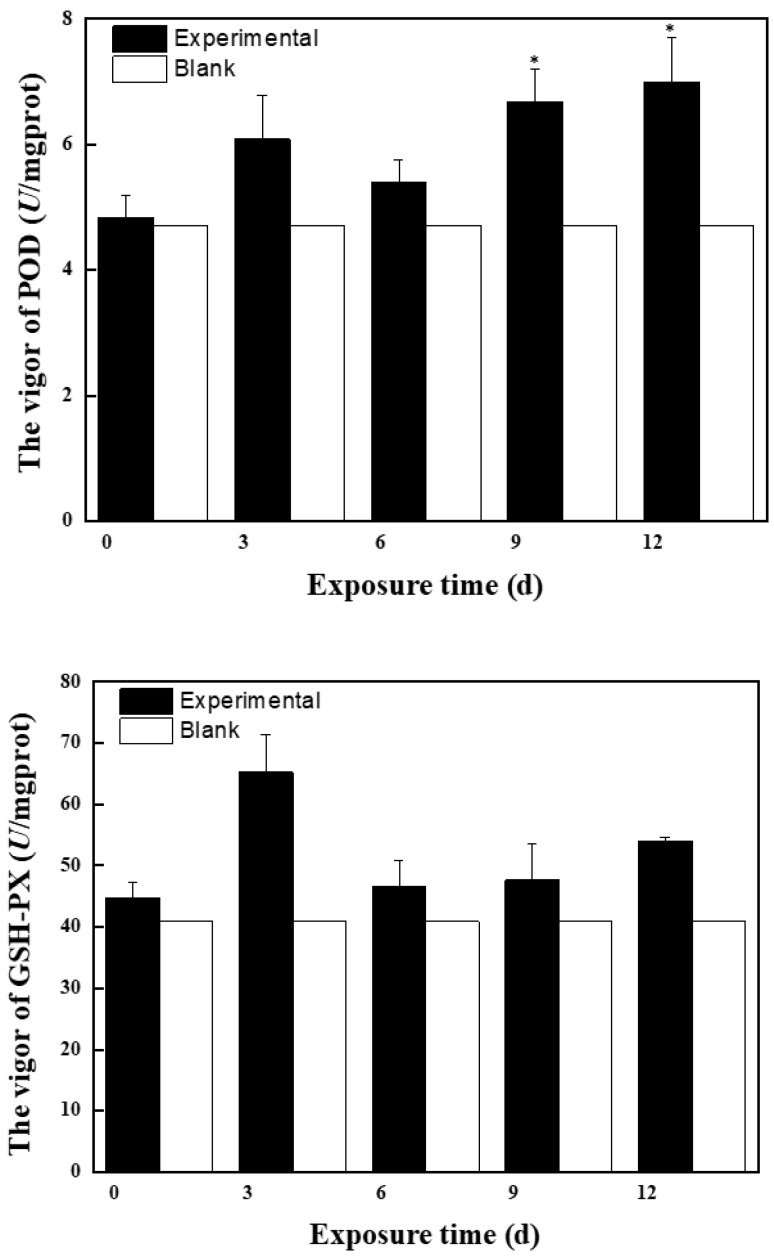

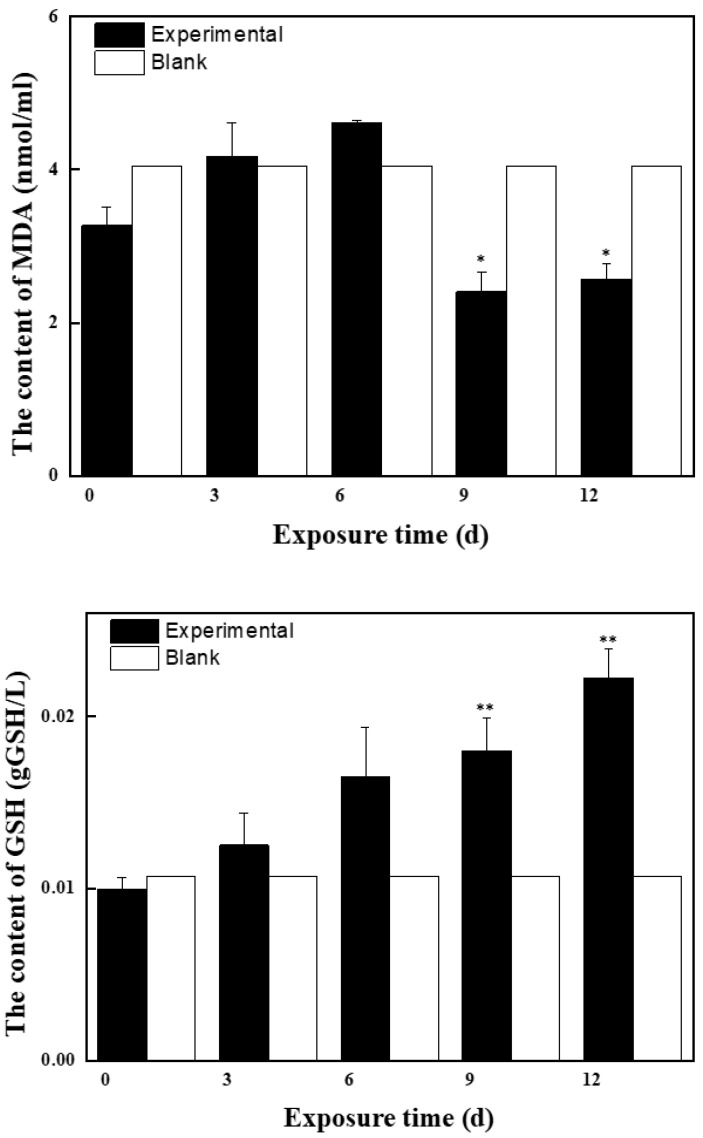
Effects of BPC and BPZ on antioxidant enzyme activities and MDA content in zebrafish (*n* = 3) within 14 days (Note: 0.01 < * *p* < 0.05; ** *p* < 0.01, compared with the control group).

Figure 2 shows the effects of BPC and BPZ on antioxidant enzyme activity and MDA content in zebrafish at the first stage. Compared with the blank group, SOD activity was inhibited within 13 days, which may be because ROS produced in zebrafish gradually increased with the increase of exposure time, and SOD decreased greatly to eliminate ROS, showing an inhibitory effect. Compared with the blank group, POD, GSH, and GSH-Px activities were induced within 13 days. POD activity was significantly induced at day 9 (*p* = 0.034) and day 12 (*p* = 0.046) (0.01 < *p* < 0.05), GSH activity was also significantly induced at day 9 (*p* = 0.006) and day 12 (*p* = 0.001) (*p* < 0.01), indicating that zebrafish can eliminate excessive free radicals by producing POD, GSH, GSH-Px, and other antioxidant enzymes so that the system can avoid oxidative damage. With the increase of exposure time, MDA content in zebrafish changed. The MDA content in the first phase reached the highest at day 7 (4.6 nmol/mL), which was higher than the control group. Higher MDA content was a stress response mechanism of oxidation that fish tissue showed due to an exogenous pollutants electrophilic group. Exogenous pollutants induced zebrafish to produce large amounts of oxygen free radicals, which in time will combine with the unsaturated fatty acid in biofilm, causing lipid peroxidation reaction and indirectly reflecting the degree of tissue cell damage.

#### 2.2.2. Effects of Mixed Solution at the Second Stage on Antioxidant Enzyme Activity and MDA Content in Zebrafish

**Figure 3:**
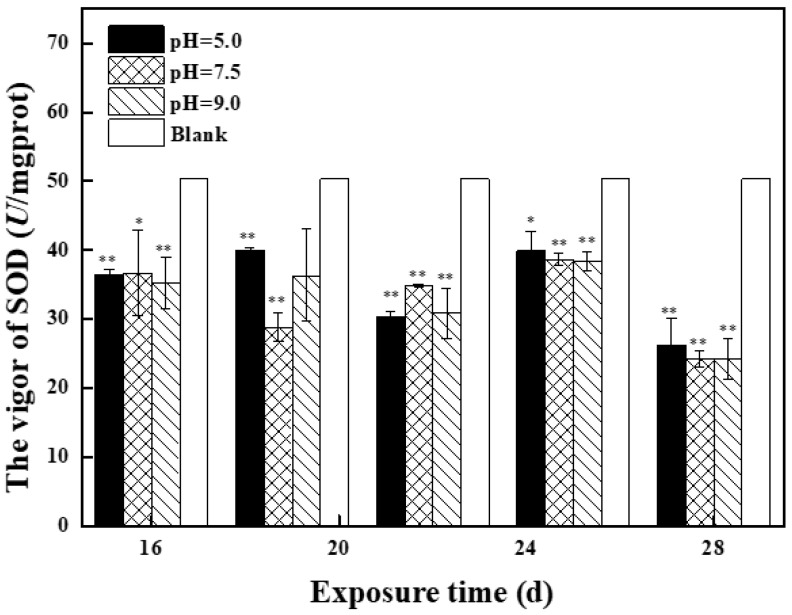

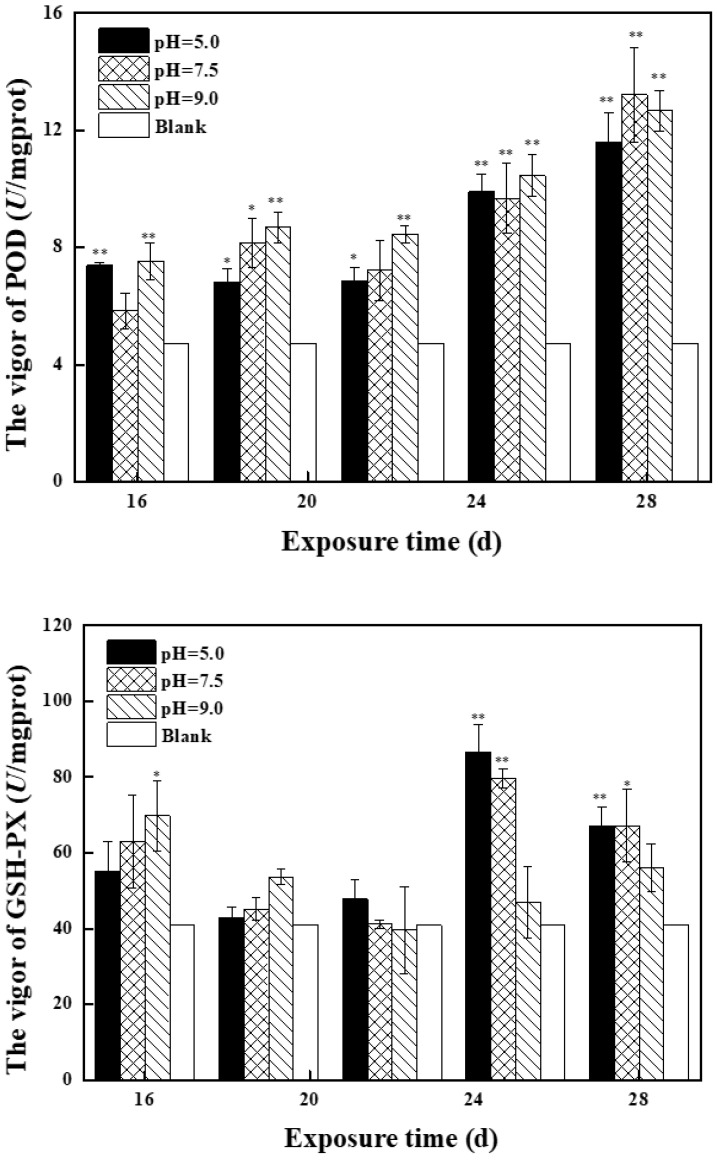

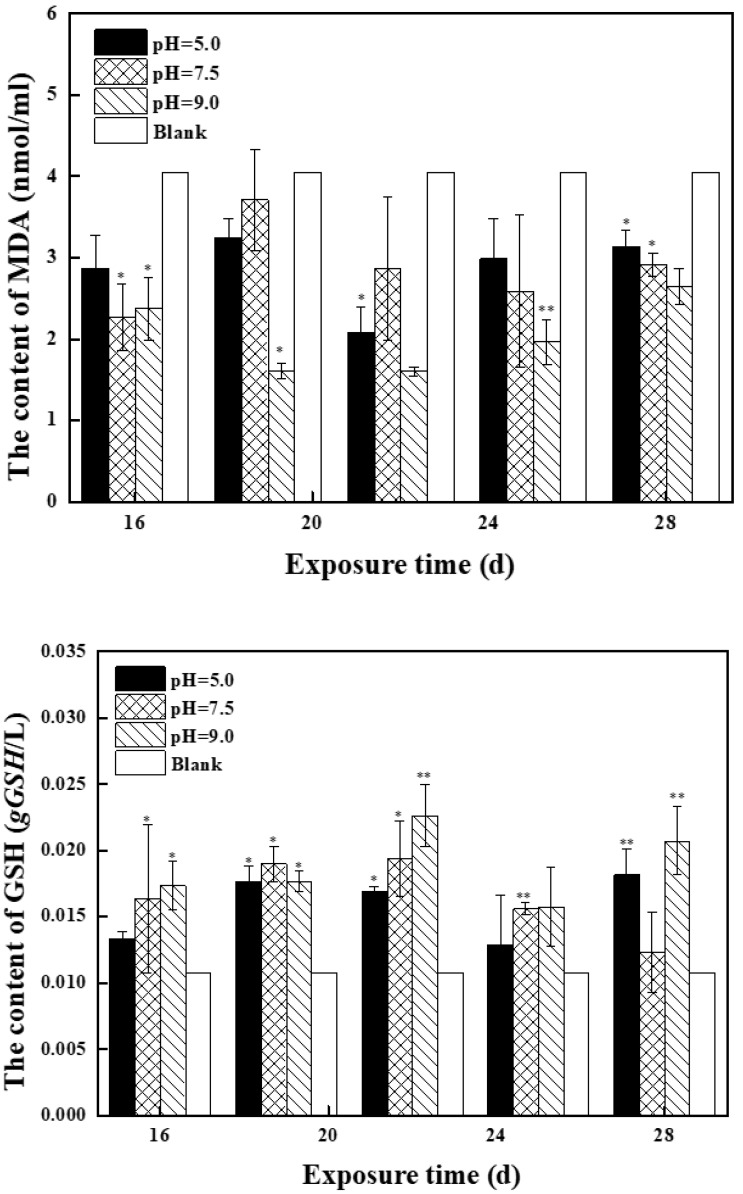
Effects of BPC and BPZ on antioxidant enzyme activity and MDA content in zebrafish (*n* = 3) under different pH conditions (Note: 0.01 < * *p* < 0.05; ** *p* < 0.01, compared with the control group).

Figure 3 shows the effects of treatment solution with different pH at different exposure times on antioxidant enzyme activity and MDA content in zebrafish in the second stage. Compared with the control group, SOD was mainly inhibited in the middle and high pH groups and reached the lowest value on day 28. On day 28, SOD activity of the three groups, with *p* values of 0.004, 0.0002, and 0.006, was significantly inhibited (*p* < 0.01) and SOD activity was similar, indicating that exposure solution caused certain oxidative damage to zebrafish, and there was no correlation with the pH value of exposure solution. Compared with the control group, the POD activity of the three groups was induced in the second stage and reached the highest value at day 28. POD activity of the low pH group was 11.6 U/mg Prot, the middle pH group was 13.2 U/ mg Prot, and the high pH group was 12.7 U/mg Prot. Compared with the control group, the activity of GSH-Px in the three groups was induced in the second stage. The maximum value of GSH-Px in the middle and low pH groups was 79.7 U/ mg Prot and 86.6 U/ mg Prot on day 25, and the maximum value of GSH-Px in the high pH group was 69.8 U/ mg Prot on day 16. It can be seen that the POD activity in zebrafish was induced similarly in the middle and low pH groups, and the induced level was higher than that in the high pH group. Compared with the control group, GSH activity in the second stage in three groups received induction, in which the pH group of zebrafish in vivo activity of GSH entrainment dropped to close to the control level after rising first, presenting normal distribution, as zebrafish oxidative stress in the body system can clear excess harmful free radicals, protecting zebrafish from oxidative damage. GSH activity was significantly induced in high (*p* = 0.043) and low pH (*p* = 0.024) groups after 22 days (0.01 < *p* < 0.05), the maximum value was reached at day 22 in the low pH group and day 28 in the high pH group. Compared with the first stage, MDA content in the three groups in the second stage changed to varying degrees, and MDA content in the middle and low pH groups increased first and then decreased, indicating that exogenous pollutants induced zebrafish to produce a large number of oxygen free radicals and timely removal. MDA content in the high pH group decreased first and then increased, but it was still lower than that in the control group, indicating that the body cells in zebrafish can still resist the attack of free radicals.

## 3. Materials and Methods

### 3.1. Chemicals and Materials

Zebrafish (type AB) (protocol number: SCXK 2016-0010) used in this experiment were males and three months old, purchased from Shanghai Jiayu Aquarium in China, with an average body length of 2.5 ± 0.5 cm and an average weight of 0.17 g. After body surface disinfection with 5% sodium chloride solution, the zebrafish were domesticated in tap water which had been dechlorinated after 72 h of aeration. The pH of the test water was 7.74–7.83. The hardness of water was 91–108 mg/L (based on CaCO_3_); the concentration of dissolved oxygen was 7.45–7.60 mg/L; the temperature was controlled at 25 ± 0.5 °C; and the time distribution was a 14:10 h day-night cycle. Zebrafish were domesticated in the laboratory for more than 7 days, during which they were fed with commercial feed twice a day, and they could not be used in the following experiments until the mortality rate within 7 days was less than 5%.

Bisphenol C (BPC) (>98.0%) and bisphenol Z (BPZ) (≥98.0%) were purchased from Shanghai Aldin Reagent Co., Ltd., Shanghai, China. Dimethyl sulfoxide (DMSO) was purchased from Shanghai Lingfeng Chemical Reagent Co., Ltd., Shanghai, China. Isotope internal standard BPA-^13^C_12_ (^13^C_12_H_16_O_2_) (99.0%) was purchased from A ChemTek, Inc., MA, USA. Trizol reagent, dATP, dTTP, dCTP, and dGTP were purchased from Thermo Scientific, Waltham, MA, USA. DNase I was purchased from New England Biotechnology (Suzhou) Co., Ltd., Suzhou, China. Ethyl m-aminobenzoate was purchased from Macklin Co., Ltd., Shanghai, China.

Glutathione peroxidase (GSH-PX) test box, glutathione (GSH) test kit, catalase (POD) test kit, malondialdehyde (MDA) test kit, total superoxide dismutase (T-SOD) test box, and total protein (TP) test kit were purchased from Nanjing Jianceng Institute of Biology, Nanjing, China. SYBR^®^ Premix Ex Taq™ II (Perfect Real Time) Kit and TaKaRa AMV Kit were purchased from TaKaRa Bio Inc., Tokyo, Japan. Ready-to-eat no-hatching harvest shrimp egg feed was purchased from Ching Yee Company, Hong Kong, China.

### 3.2. Experimental Instruments

Hardness tester (model 16900, Hach Company, Loveland, CO, USA), dissolved oxygen tester (HQ30d, Hach Company, Loveland, CO, USA), electronic analysis balance (ATY124, Shimazu Company of Japan), ultrasonic cleaning machine (UC-4600, Shenzhen Lanjie Ultrasonic Electric Co., Ltd., Shenzhen, China), ultra-pure water machine (UPR table type, Sichuan Youpu Ultra-pure Technology Co., Ltd., Chengdu, China), hand-held homogenizer (S10, Shanghai Jingxin Industrial Development Co., Ltd., Shanghai, China), UV spectrophotometer (UV-3000PC, Shanghai Meipuda Instrument Co., Ltd., Shanghai, China), six-link magnetic heating stirrer (HJ-6A, Jintan District Shuibei Science Experimental Instrument Factory, Changzhou, China), digital display constant temperature water bath (HH4, Shanghai Lichen Bangxi Instrument Technology Co., Ltd., Shanghai, China), low temperature centrifuge (model 5417R, Eppendorf, Hamburg, Germany), real-time PCR System (ABI 7500, Thermo Scientific, Waltham, MA, USA), thermal cycle (MODEL ETC-811, Beijing Dongsheng Innovation Co., Ltd., Beijing, China), gel electrophoresis and imaging system (Bio-Rad Laboratories, Inc., Hercules, CA, USA), and micro UV nucleic acid quantification system (NanoDrop ND-1000, Thermo Scientific, Waltham, MA, USA) were used.

### 3.3. Experiment Design

**Figure 4:**
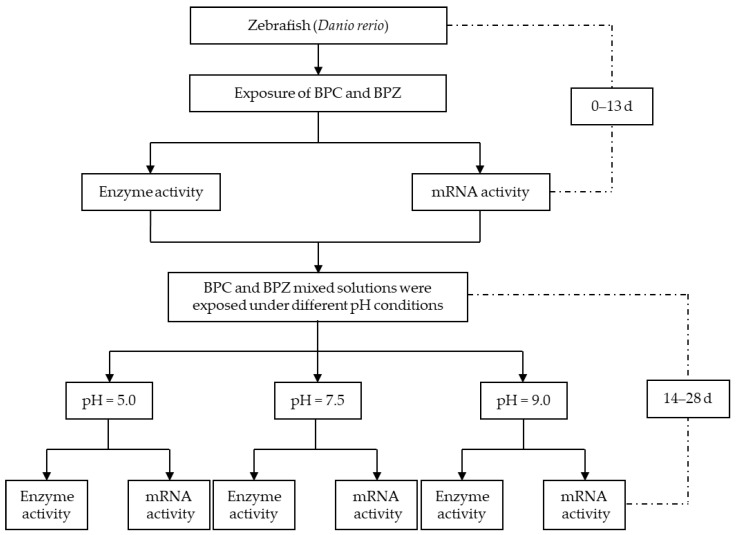
Flow chart of the experimental design in this study.

The experimental design explaining the experiments throughout the time is shown in Figure 4.

#### 3.3.1. Zebrafish Farming Method

Wild AB strain zebrafish were cultured in an independent breeding system with tap water filtered by activated carbon, UV sterilized, and automatically circulated in the system with a temperature controlled at 25 ± 0.5 °C, while maintaining a photoperiod of 14 (light): 10 (dark). They were fed twice a day with commercially available food.

#### 3.3.2. Method of Exposure

According to the authors’ previous experimental results, the semi-lethal concentrations of BPC and BPZ were 2.76 mg/L and 2.57 mg/L, respectively, and the experimental exposure concentration was 1/100 of the semi-lethal concentration [17]. In this paper, DMSO was selected as the cosolvent, and the exposure concentrations of BPC and BPZ were 0.02 mg/L. The exposure stage was divided into two stages, the first stage was 13 days, the second stage was 14–28 days. In the first stage, only 0.02 mg/L of BPC and BPZ mixed solution was added to the natural aerated water. Nine parallel experiments were repeated nine times in each group. In the second stage, by adjusting the pH value of the treatment solution, nine groups of parallel exposed solution were divided into three groups of medium and high pH, with three parallel groups in each group, and the experiment was repeated three times. Three representative pH values were selected in the experiment, namely 5.0, 7.5, and 9.0. During the experiment, 1.2 mol/L HCL solution and 2.0 mol/L NaOH solution were used to adjust the pH of the treatment solution to 5.0 and 9.0, and pH 7.5 was the same natural aeration water as the first 13 days. The exposure method was semi-static, and the treatment solution was replaced every 24 h. The pH, dissolved oxygen, and hardness values of the treatment solution were tested 2 h before and 2 h after the water change. During the experiment, they were fed once a day and fasted for 24 h before analysis and determination. During the experiment, 10 L of the corresponding concentration of experimental liquid was added to the glass aquarium, and the domesticated zebrafish were put into each exposed group with 40 fish in each group (*n* = 40). The physiological indexes in zebrafish were measured 1 d, 4 d, 7 d, 10 d, 13 d, 16 d, 19 d, 22 d, 25 d, and 28 d after exposure. As blank control, 0 d was used for comparison.

#### 3.3.3. Determination of Physiological Indicators in Zebrafish

Three tail zebrafish (*n* = 3) were randomly selected from each group at the sampling time. Zebrafish were situated in 50 mg/L of ethyl m-aminobenzoate solution for 15 min. Euthanasia of zebrafish was carried out following the Guidelines for the Euthanasia of Animals (2013) published by the America Veterinary Medical Association (AVMA), which was in accordance with the relevant requirements and principles in the animal protection and animal welfare. Zebrafish were frozen in the ice pack immediately; their surface was cleaned with cold saline, filter paper blot moisture was accurately weighed; adding the precooling 0.90% saline (group wet weight: physiological saline = 1:9), using handheld homogenate in the ice water bath machine tissue homogenate in full, made from 10% of the tissue and serum. Centrifuged at 2000 r/min at 4 °C for 10 min, the supernatant was measured for GSH level, MDA content, GSH-Px, SOD, and POD activities. The specific operation was carried out according to the instructions of Nanjing Jian-cheng Kit, and the protein content was determined by Coomassie bright blue method.

#### 3.3.4. Expression of Oxidative Stress Gene and Estrogen Receptor Gene in Zebrafish

The frozen tissue of zebrafish (*n* = 3) randomly selected from each group was quickly ground in liquid nitrogen and the powder after grinding was added with an appropriate amount of Trizol reagent. The total RNA of the sample was reversely transcribed into cDNA. Primers for each target gene were obtained from Jiangsu Hongzhong Biotech Co., Ltd. (Table 1). The PCR reaction mixture (25 μL) consisted of SYBR^®^Premix Ex TaqTM II 12.5 μL, nuclease-free water 8.5 μL, forward primer 1 μL, reverse primer 1 μL. and cDNA template 2 μL. Amplification conditions were 95 °C for 2 min, 96 °C denaturation for 10 s, 60 °C denaturation for 30 s, a total of 40 cycles, each RNA repeated 3 times, to form biological replication.

### 3.4. Statistical Analysis

Microsoft Excel 2019 software was used to analyze the obtained data, and Origin 8.5 was used for experimental mapping. The experimental results were expressed as mean ± standard deviation (mean ± SD), and the *t*-test was used to test the statistical difference between the two groups. A * *p* value < 0.05 means significant difference, ** *p* < 0.01 means extremely significant difference. Using RP17 as the reference gene, the relative expression of the target gene was calculated by 2^−∆∆Ct^ [18].

## 4. Conclusions

In the present study, it was found that the bisphenol C (BPC) and bisphenol Z (BPZ) mixture could induce an oxidative stress response and affect the expression of oxidative stress-related genes in zebrafish. Antioxidant enzymes SOD, POD, GSH, and GSH-Px as well as antioxidation-related genes CAT, SOD, and GPX could be potential biological indicators for oxidative stress detection of zebrafish. Under different pH conditions, both SOD in enzymes and genes were inhibited. GSH-Px and GPX were induced in all groups. POD and GSH were induced in a neutral environment and more strongly than in the acidic and the alkaline environments. The MDA content in the exposed group was lower than that in the blank control group, indicating that free radicals in zebrafish were effectively eliminated. Thus, BPC and BPZ could not only affect the antioxidation system but also cause an estrogen effect on zebrafish. Further studies need to be performed to assess the toxicities of bisphenol analogs, helping to provide evidence for toxicity mechanisms of bisphenol analogs to aquatic organisms.

## Figures and Tables

**Table 1:** Primer sequences of candidate reference genes.

Gene Symbol	Accession NO.	Primer Sequence (5′-3′)	Product Length (bp)
*β-actin*	AF025305.1	F: CGAGCTGTCTTCCCATCCA R: TCACCAACGTAGCTGTCTTTCTG	86
*rp17*	NM_213213644.2	F: CAGAGGTATCAATGGTGTCAGCCC R: TTCGGAGCATGTTGATGGAGGC	119
CAT	AF170069.1	F: CTCCTGATGTGGCCCGATAC R: TCAGATGCCCGGCCATATTC	126
SOD	BX055516	F: GTCCGCACTTCAACCCTCA R: TCCTCATTGCCACCCTTCC	217
GPX	AW232474	F: AGATGTCATTCCTGCACACG R: AAGGAGAAGCTTCCTCAGCC	94
ER*α*	AF268283	F: CCC ACA GGA CAA GAG GAA GA R: CCT GGT CAT GCA GAG ACA GA	250
ERβ1	AJ414566	F: GGG GAG AGT TCA ACC ACG GAG R: GCT TTC GGA CAC AGG AGG ACG	89

## Data Availability

The authors declare that all data generated or analyzed during this study are included in the published article.

## Sample Availability

Samples of the compounds are available from the authors.
